# Improving mental health literacy of educational professionals: feasibility and preliminary effectiveness of an adapted intervention LEARN-NL

**DOI:** 10.1186/s12889-025-23836-4

**Published:** 2025-09-30

**Authors:** Janne M. Tullius, Bas Geboers, Sijmen A. Reijneveld, Andrea F. de Winter

**Affiliations:** Department of Health Sciences, University Medical Center Groningen, University of Groningen, PO Box 196, Groningen, 9700 AD The Netherlands

**Keywords:** Mental health literacy, Internet-based intervention, Feasibility, Educational professionals, Adolescent mental health, Acceptability, Implementation, Preliminary effectiveness

## Abstract

**Background:**

The high prevalence rate of adolescent mental health problems is a widespread global concern. Educational professionals in secondary education are in a prime position to address this but often lack knowledge and competencies regarding mental health (‘mental health literacy’) for this. We developed LEARN-NL, an internet-based self-paced intervention that is promising to improve mental health literacy among educational professionals. The aim of this study was to evaluate the feasibility of the LEARN-NL intervention in terms of acceptability, implementation, and preliminary effectiveness.

**Methods:**

We performed a mixed methods feasibility study with *n* = 29 educational professionals in the Netherlands. Questionnaires on educational professionals’ mental health literacy, assessed by the Mental Health Literacy and Capacity Survey for Educators (MHLCSE) and subscales of the Knowledge and Attitudes to Mental Health Scales (KAMHS), were administered before and after the intervention to assess preliminary effectiveness of LEARN-NL. We further interviewed participants (*n* = 9) after completing the intervention to assess acceptability and implementation. Quantitative data were analyzed pre- vs. post-training, qualitative data with thematic analysis.

**Results:**

The LEARN-NL intervention was perceived by the educational professionals that completed the training as acceptable and useful, especially in terms of three main themes: (1) Participant satisfaction, (2) rich diversity of topics covered, and (3) better mental health literacy of educational professionals to support adolescents. LEARN-NL could be implemented as planned and we identified two main themes: (1) Format, and (2) conditions for broad implementation and impact. We further found preliminary effectiveness of LEARN-NL intervention in increasing mental health literacy of educational professionals, in terms of self-perceived awareness, knowledge, and comfort, and decreased stigma (*p* < 0.001).

**Conclusions:**

LEARN-NL is feasible as it is acceptable and implementable as planned for improving mental health literacy of educational professionals and shows promising preliminary effectiveness. Larger-scale effectiveness studies should be performed to further investigate the intervention.

**Supplementary Information:**

The online version contains supplementary material available at 10.1186/s12889-025-23836-4.

## Background

Up to 25% of all young people between the ages 10 and 18 experience mental health problems worldwide [1, 2]. When left unidentified and untreated, mental health problems during adolescence can have a long-lasting detrimental impact on young individuals’ lives [3]. Adolescents grappling with mental health problems are at a higher risk of experiencing physical health problems, and social, academic, and personal limitations along with long-term mental health problems [4, 5]. Improved strategies are required to intervene in this negative trend.

Schools can play an important role in mental health promotion, identification of mental health problems, and providing appropriate support resources [6]. Especially educational professionals (e.g., teachers, counsellors, and school support staff) are in a prime position to identify signals of mental health problems and support adolescents in their help-seeking decisions and behaviors [7–9]. They also can play an instrumental role in the creation of a classroom environment where students feel safe discussing their mental health and engaging in productive collaborations with other stakeholders to support student mental well-being [8]. However, many educational professionals don’t feel adequately equipped to address their students’ mental health concerns [10–13].

Promoting mental health literacy (MHL) among educational professionals holds a large potential in providing them with the necessary knowledge and skills to identify and address adolescent mental health problems [9, 14]. MHL has been acknowledged as an essential basis for educational professionals in promoting adolescents’ mental health but is mostly overlooked in their initial training programs [8, 15–17]. The concept MHL encompasses the essential knowledge and skills regarding mental health, or the capacity to “understand how to obtain and maintain positive mental health; understand mental disorders and their treatments; decrease stigma related to mental disorders; and, enhance help-seeking efficacy (knowing when and where to seek help and developing competencies designed to improve one’s mental health care and self-management capabilities)” [18].

In recent years, attention has grown for interventions aimed at enhancing the MHL of educational professionals in secondary education, and these efforts have shown promising results [19, 20]. MHL interventions for educational professionals typically aim to enhance mental health knowledge (e.g., knowledge and recognition of mental disorders), stigma reduction and help-seeking behavior through educational lectures, interactive seminars, paper materials, online materials, role plays, situational experiences, games, and practical assignments [21]. Enhancing educational professionals’ MHL through interventions in the school setting has shown positive effects not only for their improved knowledge, reduced stigmatizing attitudes, and supporting behaviors but also on student mental health literacy [19, 22, 23]. One such intervention is the ‘LEARN’ intervention, a Massive Open Online Course (MOOC) originally developed by mentalhealthliteracy.org in collaboration with three Canadian Faculties of Education, targeting (pre-service) educational professionals in a digital environment [20]. This intervention has received positive evaluations in the Canadian context with regard to improvements in knowledge, stigma reduction, and enhancement on help-seeking intentions among educational professionals and, by extension, their students [20, 24].

In earlier work, we described a systematic process to adapt the ‘LEARN’ intervention to the Dutch setting following the Intervention Mapping Adapt protocol [25]. This resulted in the MHL intervention ‘LEARN-NL’ [20], taking place in the Dutch language in the Netherlands. As the next step before investigating effectiveness, its feasibility should be studied in the new cultural setting. More specifically, the users’ acceptability ought to be assessed, potential implementation challenges identified, and preliminary claims on its effectiveness made [26–28]. Therefore, the aim of the current study was to evaluate the feasibility of the LEARN-NL intervention in terms of acceptability, implementation, and preliminary effectiveness.

## Methods

### Study design

We performed a mixed methods study combining qualitative and quantitative methods to assess the feasibility of the LEARN-NL intervention in terms of acceptability, implementation, and its preliminary effectiveness for educational professionals in secondary education. *Acceptability* regards how the intended targeted individuals react to the intervention; *implementation* concerns the extent, likelihood, and manner in which an intervention can be fully implemented as planned and proposed, and *preliminary effectiveness* regards whether the intervention shows promise of reaching its intended goals with the intended population [28]. This study was performed between April and December 2023.

This study was performed in accordance with the Helsinki Declaration, and informed consent was obtained from all participants. The study was deemed exempt from human subjects’ review (non-WMO study) and a waiver of ethical approval was granted by the Medical Ethics Review Board of the University Medical Center Groningen (METc UMCG) (no. M20.252893).

### Participants

A total of 29 participants were included in this study, which is considered standard practice for feasibility or pilot studies [29]. Participants were recruited through school organizations in the Netherlands and snowballing techniques. Additionally, information meetings were conducted to provide potential participants with insights into the training program and the research process. Twenty-six individuals attended the information meetings. Participants were eligible to participate if they worked with adolescents in a Dutch secondary school (e.g., educator, support staff, orthopedagogue, care staff).

### The LEARN-NL intervention

The LEARN-NL intervention consists of an online mental health literacy training for educational professionals in secondary education in the Netherlands. It is designed as a comprehensive online self-study course that offers participants an opportunity for reflection and engagement through interactive activities, videos, and practical tools and tips. The training is structured into six modules, each addressing crucial aspects of mental health literacy and mental health support for adolescents in secondary education: (1) Basic concepts on mental health literacy and mental health, (2) Understanding stress and the stress response, (3) Stigma of mental health disorders, (4) The development of the adolescent brain, (5) Most common mental health disorders of adolescents, (6) Seeking help and providing support. Each module takes about 1.5 h to complete after which a short quiz can be filled in to test one’s own understanding of the materials. Participants have the flexibility to complete the modules at their own preferred pace and from any location using electronic devices (e.g., tablet, laptop, smartphone).

LEARN-NL is based on a Canadian evidence-based intervention (‘LEARN’) and was translated and culturally adapted for the Dutch population using the Intervention Mapping Adapt framework [25]. The adaptation process was performed in collaboration with educational professionals, adolescents, and mental health and care experts to tailor the training to the needs of the end-user. In the adaptation process, besides translations of the materials and adaptations specific to the Dutch cultural context, main adaptations included the omission of a module on treatments and content that endorsed mainly the biological perspective of mental disorders as well as a reorganization of the order of modules (Module 7 becoming Module 2 in LEARN-NL). A more detailed description of the intervention can be found in Supplementary Materials. The systematic intervention development following the IM Adapt method is reported elsewhere [25].

### Procedure

For the feasibility evaluation of LEARN-NL, participants completed questionnaires before and after receiving the intervention and were invited to participate in interviews. First, participants were asked to fill in the first questionnaire (T0), which was sent to them via email. They could fill it out either on their own or at the end of an information meeting if they attended. Second, they received the online training (LEARN-NL) via email and were asked to complete the training within six weeks. If participants hadn’t completed the training within that time frame, they received a reminder and up to three additional weeks to complete the training. We considered the training completed when the participants had completed at least three of the six modules. Third, after completion of the training, participants received the same questionnaires as prior to the training including a few additional questions (T1; see ‘Outcome measures’) as well as an invitation for an interview with one of the researchers. Quantitative data were collected and managed using the REDCap electronic data capture application [30]. All participants that completed the training received a certificate of participation; no other incentives were provided.

Finally, interviews were held with the participants who agreed to partake. The interviews were semi-structured and guided by an interview guide. The interviews were led by the first author and lasted between 30 and 60 min. All interviews were recorded with an audio recorder. Interviews were held between one to three weeks after the participants had completed the training. Data collection continued until data saturation was reached. Data saturation was reached when new data collected did not yield any new information [31].

### Outcome measures

#### Acceptability & implementation

Acceptability and implementation of the intervention were assessed by conducting interviews with the participants. The interview guide was established based on the Measurement Instrument for Determinants of Innovations (MIDI) [32] and was structured to first address the participants’ acceptability. For example, the participants were asked about the benefits they had had from the training (Determinant 8). They were also asked what modules they liked best and were of the most value, which one they liked the least, and whether the training was tailored to their needs and those of their students. Then they were asked more specifically about the implementation, for example how they perceived their ability to apply the newly acquired knowledge and skills in practical situations in the future (Determinant 16). They were also asked how exactly they had followed the training, how they had combined it with daily activities, and how they perceived the format and methods of the training. The complete interview guide can be found in the Supplementary Materials.

Acceptability of the intervention was also assessed quantitatively at follow-up by asking to rate the training overall and its contributions on a scale from 1 to 10 to the following seven domains: (1) Knowledge about mental health, mental disorder, stress response, (2) Own stress management, (3) Supporting stress management of students, (4) Knowledge about mental disorder and mental health problems, (5) Supporting mental health problems and mental disorder in students, (6) Identifying mental health problems in students, and (7) Insight into the aid process, resources and possible healthcare professionals. Ratings of 10 were labelled “The training contributes extremely” while ratings of 1 were labelled “The training contributes nothing at all”.

#### Preliminary effectiveness

Preliminary effectiveness was assessed by measuring participants’ MHL before and after completing the intervention. We measured MHL with two scales: the Mental Health Literacy and Capacity Survey for Educators (MHLCSE) and the Knowledge and Attitudes to Mental Health Scales (KAMHS). Also, some general MHL items were included.

##### Mental Health Literacy and Capacity Survey for Educators (MHLCSE)

The MHLCSE holds three sub-scales: Awareness (6 items), Knowledge (5 items; measured subjectively) and Comfort (4 items). Participants rate statements on a 5-point Likert scale ranging from 1 = ‘not at all aware’, ‘not knowledgeable’ or ‘not comfortable’ to 5 = ‘very aware’, ‘very knowledgeable’ or ‘very comfortable’, respectively. Internal consistency of the MHLCSE has been found to be high (Cronbach’s alpha = 0.88–0.91) [12]. The scale was translated to the Dutch language for the purposes of this study. The translation was done through a forward-backward translation process (English to Dutch, Dutch to English) by the first author (JMT) and a research assistant. Discrepancies were resolved through discussion and the final version of the Dutch version was discussed with the two co-authors BG and AFdW. At T1, it was additionally asked to what extent the intervention had contributed to the self-rated awareness, knowledge, and comfort of the participants on a 5-point Likert scale ranging from 1 = ‘not at all’ to 5 = ‘very much’

##### Knowledge and Attitudes to Mental Health Scales (KAMHS)

The KAMHS was used to measure the MHL domains ‘knowledge about mental health’, ‘stigma’, and ‘self-stigma’. The KAMHS is a reliable multifaceted self-report questionnaire measuring MHL in adolescents through the six domains of MHL [33]. Internal consistency of the KAMHS has been found to be fair to good (McDonald’s omega = 0.53–0.80) [33]. For the purposes of this study, only the subscales “knowledge about mental health” (12 items; measured objectively), “(lack of) stigma” (6 items) and “(lack of) self-stigma” (6 items) were used. A validated Dutch translation of the scale was used [34] and adapted to the adult educator population. For example, the item “I would not like to be in the same classroom as someone with a mental disorder” was adapted to “I would not want to have a student with a mental disorder in my class” and the item “If I had a mental health problem, I would be happy to tell my teacher or school counsellor” was changed to “If I had a mental health problem, I would be happy to tell my superior”. Respondents were asked to rate statements on a five-point Likert scale (strongly agree, agree, don’t know, disagree, strongly disagree). Several items are reverse scored and average scores for each subscale are computed, resulting in an average score between 0 and 4, with higher scores indicating better knowledge and lower stigma.

##### General item*s*

In addition, participants were asked to rate four overarching statements related to their mental health competencies and support at school on a 5-point Likert scale (strongly disagree, disagree, no opinion, agree, strongly agree): (1) ‘I am fully trained as a professional to deal with students who have a mental health problem or mental health disorder.’, (2) ‘I am confident in my skills to identify mental health problems in students.’ (3) ‘Access for students to help resources at school needs to be better.’ (4) ‘It is important that educational professionals have insights into the mental health problems that students can experience.’

#### Background characteristics

Background characteristics included age, gender (male, female, other), school level in which the professional worked (lower secondary, intermediate secondary, higher secondary, special education), work function (educator, care coordinator, support staff, other), experience working in education (in years), and previous exposure to mental health problems (e.g., through students, colleagues, private environment, own experience).

### Data analysis

We first described the background characteristics of the sample. Next, the qualitative data from the interviews were used to evaluate the acceptability and implementation of the intervention. The interviews were transcribed and coded by the first author (JMT), in consultation with BG and AdW. JMT and BG coded two transcripts, after which consensus was reached through comparison and discussion of the results. Afterwards, JMT coded the rest of the transcripts. Codes were derived from the data and a codebook was developed which was refined throughout the coding process. A thematic analysis for both acceptability and implementation was done, using the structured interview guide as a thematic foundation to guide the categorization of main themes [35]. Main themes and codes were discussed with all co-authors before finalizing the analysis and results. The qualitative data were managed, processed, and analyzed using Atlas.ti computer software (Version 9) [36].

The quantitative data from the questionnaires were used to evaluate acceptability and preliminary effectiveness of the intervention. We assessed the acceptability of the intervention at T1 using descriptive statistics. We then assessed the preliminary effectiveness of the LEARN-NL intervention by comparing pre- and post-training scores of the MHCLSE and KAMHS subscales using paired samples t-test. Data cleaning and handling was performed by the first author of this study. The data were checked regarding the normality of their distribution with Skewness and Kurtosis tests. Data that were not normally distributed were normalized through square root transformation. All analyses were carried out using IBM SPSS Version 26. Results were considered statistically significant at *p* < 0.05.

## Results

**Fig. 1:**
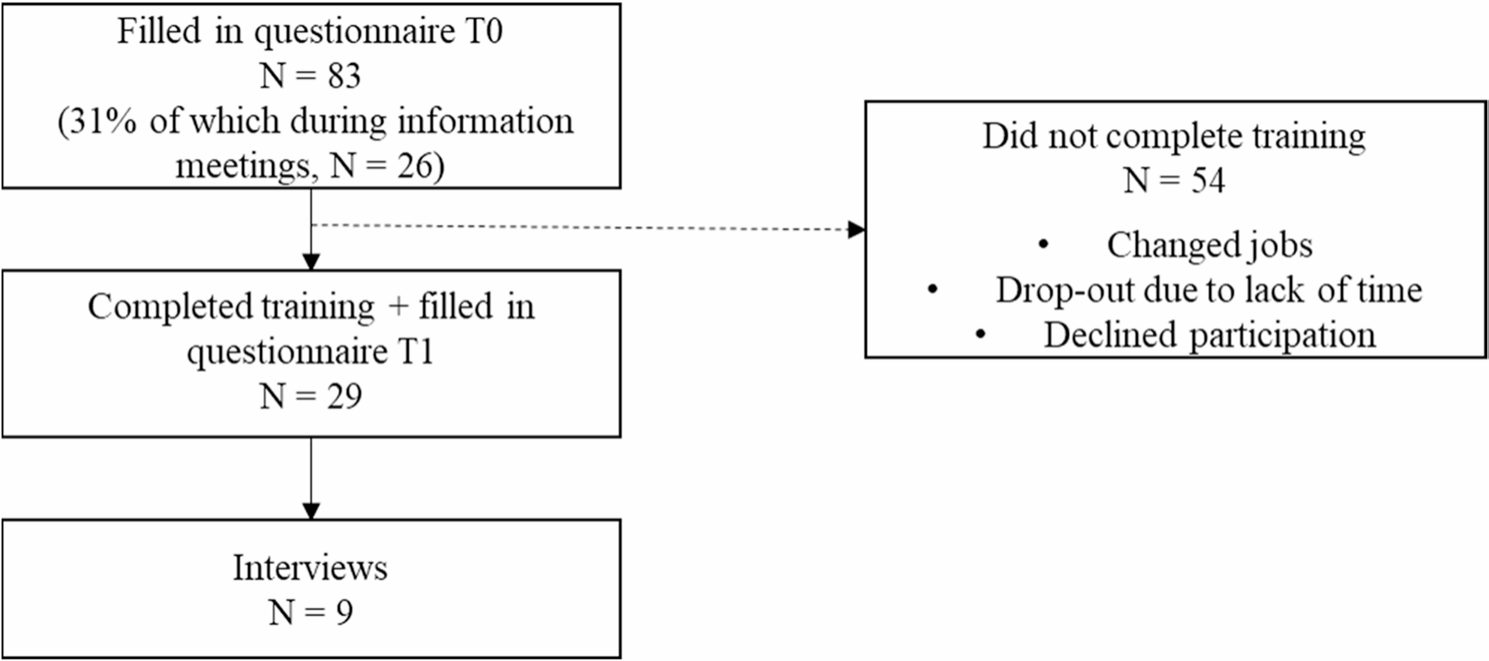
Participant flow chart of completers and non-completers

Twenty-nine educational professionals completed the training and filled in the second questionnaire (T1). Only one of the 29 completers completed fewer than six modules, finishing only four modules. Completing the training took the participants five to eight weeks. Nine participants took part in the interviews. Fifty-four other educational professionals filled in the questionnaire prior to the training (T0) and had intended to participate in the training but did not complete the training (65%). Forty-three of these non-completers (80%) have never started the training at all. For an overview of the participant flow, see Fig. 1.

### Demographics

The study sample is described in Table 1 (*n* = 29). Most participants worked as educators (72.4%) and had a role as mentors (72.4%). Most of the participants (82.8%) identified as female. Of the total sample, 69% worked at higher secondary school level, 55.2% worked at intermediate secondary education, and 51.7% worked at the lower vocational education level. The demographics of the participants are comparable to the people who signed up but had not completed the training (non-completers), even though the non-completers were on average younger and had less work experience.

**Table 1:** Participant demographics of both completers and non-completers (Chi-square test for p-values)

	Total *N* = 29 (completers)	Total *N =* 54 (non-completers)	Chi^2^ (df)	*p*
Age *M* (SD)	44.52 (10.6)	39.62 (11.7)		0.06
Gender *n* (%)			0.02 (1)	0.57
Female	24 (82.8)	44 (81.5)		
Male	5 (17.2)	10 (18.5)		
Non-binary or other	-	-		
Position *n* (%)			2.76 (4)	0.68
Educator	21 (72.4)	38 (70.4)		
Orthopedagogue	-	1 (1.9)		
Support staff	-	3 (5.9)		
Care coordinator	3 (10.3)	3 (5.6)		
Social worker	-	-		
Other (e.g., education assistant, administrative staff, supervising role)	5 (17.2)	9 (16.7)		
Role as mentor (% yes)	21 (72.4)	36 (66.7)	0.49 (2)	
School work experience n (%)			10.05 (2)	
0 to 5 years	3 (10.3)	24 (44.4)*		
5 to 15 years	14 (48.2)	17 (31.5)		
Longer than 15 years	12 (41.4)	13 (24.1)		
Work with school level^1^ *n* (%)				
Higher secondary	20 (69.0)	40 (74.1)	0.25 (1)	0.40
Intermediate secondary	16 (55.2)	38 (70.4)	1.92 (1)	0.13
Lower secondary	14 (51.7)	34 (63.0)	0.99 (1)	0.22
Special education	1 (3.4)	1 (1.9)	0.20 (1)	0.58
Work with school grade^1^ *n (%)*				
1 st grade	17 (58.6)	27 (50.0)	0.56 (1)	0.30
2nd grade	18 (62.1)	31 (57.4)	0.17 (1)	0.43
3rd grade	20 (69.0)	43 (79.6)	1.17 (1)	0.21
4th grade	18 (62.1)	34 (63.0)	0.01 (1)	0.56
5th grade	14 (48.3)	23 (42.6)	0.25 (1)	0.40
6th grade	11 (37.9)	16 (29.6)	0.59 (1)	0.30
Has been exposed to mental health problems^1^ (% yes)				
Through students	26 (89.7)	38 (70.4)*	3.98 (1)	0.04
Through colleagues	19 (65.5)	25 (46.3)	2.80 (1)	0.07
Through own experiences with mental health problems	2 (6.9)	12 (22.2)	3.16 (1)	0.07
Through friends, family, acquaintances	20 (69.0)	36 (66.7)	0.05 (1)	0.52
Through media, movies, series	11 (37.9)	24 (44.4)	0.33 (1)	0.37

^*1=*^ Multiple responses possible; df = degrees of freedom; * = statistically different,* p-value = < 0.05*

### Acceptability

#### Interviews

**Fig. 2:**
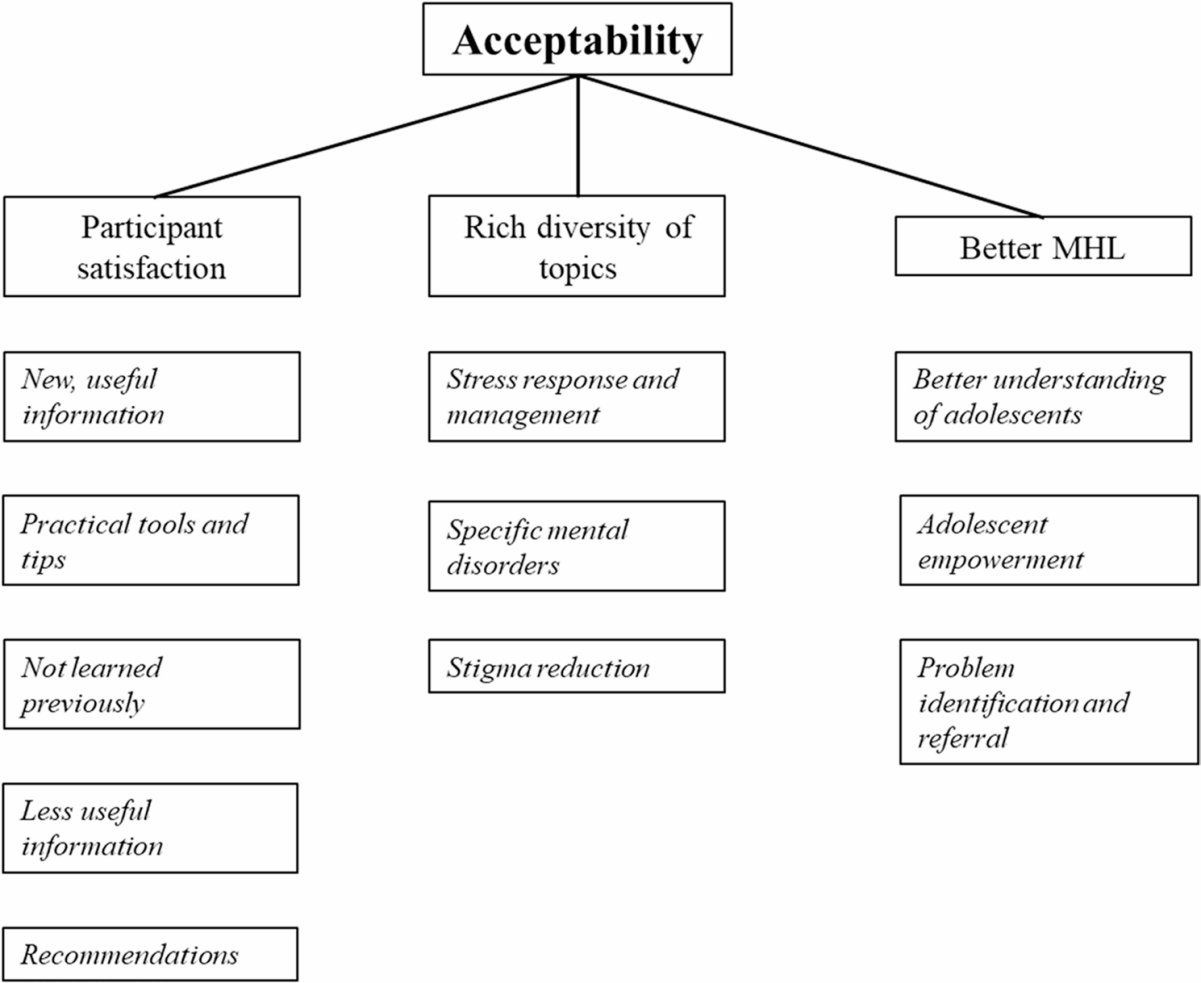
Overview of themes and subthemes on acceptability

The interviews yielded three main themes regarding the acceptability of the intervention: (1) Participant satisfaction, (2) Rich diversity of topics covered, and (3) Better mental health literacy of educational professionals to support adolescents. Some subthemes were also identified. Figure 2 displays an overview of the themes and subthemes. Quotes supporting these themes are displayed in Supplementary Materials.

***Participant satisfaction. ***All participants were positive about the intervention and their acquired mental health knowledge and competencies. We were able to identify five subthemes: (1) New, useful information, (2) Practical tools and tips, (3) Not learned previously, (4) Less useful information, and (5) Recommendations to the content.

***New, useful information***. Participants acknowledged that they gained new important information and insights through the training and highlighted the degree to which the newly acquired knowledge may support their tasks as educational professionals. They found the intervention to be relevant and in line with the difficulties they often face in the school context concerning the mental health problems of adolescents (e.g., somberness of students, lack of basic knowledge of and communication skills regarding mental health problems and understanding of diagnosed mental disorders and its effects on classroom behavior).

***Practical tools and tips***. Various participants appreciated the practical tools and tips that the intervention provided, mentioning that they were useful and easy to apply in their daily work as educators. This related especially to tips regarding communication with students or parents. Also, participants appreciated the practical tips on how to deal with certain situations (e.g., stigmatizing comments, a gut feeling that ‘something isn’t right’) and what help services to turn to if needed.

***Not learned previously***. One of the main reasons for participants for finding the intervention useful was that it included information that they had not previously encountered during their own training or professionalization opportunities. Specific information on mental health and mental disorders had rarely been addressed in previous training or professionalization. Many participants mentioned that it would be useful to include some of the modules or more mental health training in the teacher education curricula.

***Less useful content***. Some participants also mentioned modules or topics that were less useful to them, often because they already had some knowledge or experience of them, for example how to find help sources at school or communication strategies with students and parents. A few participants said that the training did not help them to better identify mental health problems in adolescents, often due to the fact that they worked as educational support staff and as such only encountered adolescents when a diagnosis had already been made.

***Recommendations about the content***. While the majority of participants indicated that they had not missed any particular topic, a noteworthy suggestion emerged: the inclusion of a future module addressing the management of aggressive or externalizing behavior exhibited by students and effective strategies for handling such situations.

##### Rich diversity of topics covered

Rich diversity of topics covered Many participants mentioned that they appreciated the rich diversity of topics in the training (e.g., positive mental health, terminology, mental health disorders, effects on academic performance). Participants also highlighted different modules and topics from the training as particularly interesting and applicable to their individual needs. Many participants expressed a heightened interest in the modules that were previously less familiar to them prior to the training. This main theme could be divided into three subthemes: (1) Stress response and management, (2) specific mental disorders, and (3) stigma reduction.

***Stress response and management***. Most educational professionals particularly found the content on stress management and the positive approach to stress meaningful to their work with adolescents in school. Many found this a topic that was most applicable and universal to all of their students and that could be easily put into practice right away, by providing education to their students on this topic, possibly aiding in preventing mental health problems.

***Specific mental disorders***. Some participants found it very useful to learn more about the different types of specific mental disorders as they hadn’t done so previously. They mentioned that their knowledge extended mainly to disorders such as depression or ADHD. Some also said they found it interesting to learn about the difference between a mental disorder and a mental health problem.

***Stigma reduction***. Others found the most value in the module on stigma as from their perspective this is an underrepresented factor that is rarely addressed or thought of. Some participants recognized the significance of understanding how language can influence attitudes towards people with mental health problems or disorders, as well as the crucial role that educational professionals can play in addressing this.

##### Better mental health literacy of educational professionals to support adolescents

All participants mentioned that they have acquired knowledge and competencies through the training that can lead to several improved outcomes for adolescents. Those could be divided into the three subthemes: (1) Better understanding, (2) adolescent empowerment, and (3) problem identification and referral to help services.

***Better understanding of adolescents***. All participants agreed that the intervention has the potential to make adolescents feel more understood and heard by educational professionals. They noted that the training may help them in developing more empathy and understanding for the problems and challenges adolescents deal with. Having more educators around them who are trained in mental health-related topics may increase the number of trusted adults to whom adolescents can turn.

***Adolescent empowerment***. Many participants said they had learned new information and strategies that can empower students to take care of their own mental health and manage stress levels, for example by teaching them relaxation techniques or changing their mindset about stress to perceive it as helpful rather than harmful.

***Problem identification and referral to help services***. A few participants pointed out that the newly acquired information may lead to a better usage of help services of adolescents. They mentioned that through the intervention, educational professionals might be able to deal with smaller problems and challenges of adolescents (e.g., test anxiety) and support them adequately rather than informing other help sources right away. Simultaneously, they might be better equipped to pick up on signals of mental health problems and make an accurate estimation of whether additional help services are needed and support the referral process.

#### Questionnaires

Table 2 shows the ratings of the intervention by the participants. Participants on average rated the training overall with 7.61, on a scale from 1 (very poor) to 10 (excellent). The Majority of domains of the intervention received a rating of 7 or higher. Only ratings for contribution to own stress management (*M* = 6.24, SD = 1.94) and insights into care processes (*M* = 6.96, SD = 1.22) were lower.

**Table 2:** Ratings of intervention (*N* = 26)

Item text On a scale from 1–10*:	Mean (SD)	Range
How do you rate the VDK-training overall?	7.64 (0.64)	7–9
To what extent does the training contribute to improving knowledge (about mental health, mental disorder, stress response, etc.)?	7.88 (0.88)	6–10
To what extent does the training contribute to improving your stress management?	6.24 (1.94)	2–9
To what extent does the training contribute to supporting stress management of your students?	7.08 (1.19)	4–10
To what extent does the training contribute to your knowledge about mental disorder and mental health problems?	7.87 (1.10)	6–10
To what extent does the training contribute to supporting the mental health problems and mental disorder in students?	7.21 (1.18)	4–9
To what extent does the training contribute to the improvement of identifying mental health problems in students?	7.40 (1.16)	5–10
To what extent does the training contribute to improved insight into the aid process, resources and possible healthcare professionals?	6.96 (1.22)	3–9

* 10 = Contributes extremely; 1 = Contributes nothing at all

### Implementation

#### Interviews

**Fig. 3:**
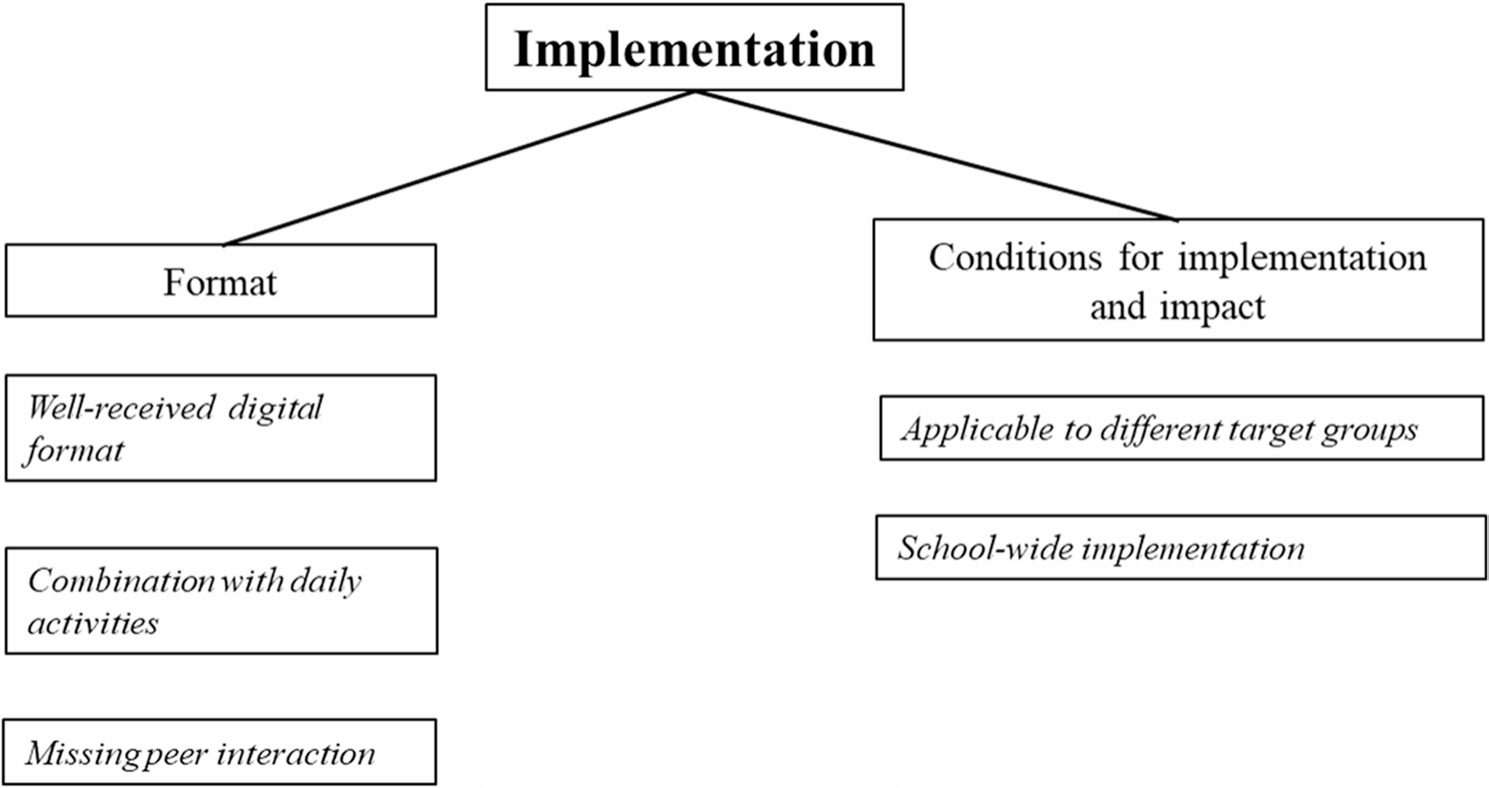
Overview of themes and subthemes on implementation

The interviews yielded two themes that were perceived as contributors, and in some cases as barriers, for the implementation of the intervention: (1) Format, and (2) conditions for broad implementation and impact. Some subthemes were identified as well. An overview of the themes and subthemes is presented in Fig. 3. Quotes supporting these themes are displayed in Supplementary Materials.

##### Format

The training’s format was one of the main factors that was mentioned by the participants as a mainly contributing factor for adequate implementation. We could divide this theme into three subthemes: (1) Well-received digital format, (2) Combination with usual activities, and (3) Missing peer interaction.

***Well-received digital format***. Most participants were very positive about the digital format of the training and said that they preferred such digital learning methods. Many participants mentioned that they liked the combination of theoretical knowledge, video clips, activities, and quizzes in the digital format. However, for some participants the digital format was seen as a barrier to complete the training as it required a great deal of discipline and task management.

***Combination with daily activities***. Many participants said that the format of the training provided greater flexibility of working on the modules at any time anywhere and at their own pace rather than having to attend group meetings in person. This allowed them to combine the training with their regular daily activities and tasks. However, the challenge of finding dedicated time for completing the training emerged as another crucial factor influencing implementation. Participants often found it difficult to complete the training during busy times of the school year. While they appreciated the flexibility of the training, completing it often found itself consistently relegated to the bottom of their priority list: Numerous participants mentioned that they completed the training on non-working days, such as weekends or holidays, due to time constraints on their working days. However, those participants said they also preferred doing it this way.

***Missing peer interaction***. Despite the flexibility and self-paced learning provided by the training, several participants expressed a desire for more interaction with peers to discuss the acquired information and practice communication strategies.

##### Conditions for broad implementation and impact

Factors were mentioned that may influence the broad implementation and impact. These can be divided in two subthemes: (1) Applicable to different target groups, and (2) School-wide implementation.

***Applicable to different target groups. ***Many participants mentioned that they considered the intervention not only to be useful to educators, but also to other target groups, for example school administration, management staff and educators in training. It was mentioned that the demands of individuals working in schools and with adolescents are changing, for example due to the Inclusive Education Act (Wet Passend Onderwijs) which gives schools more responsibility of care for adolescents with specific needs. Therefore, also educator training should include topics related to mental health to meet this demand.

***School-wide implementation. ***When asked how participants would like to see the topic of mental health literacy and mental health support play a role further in their schools, different aspects were mentioned. One participant said that they hoped that as many colleagues as possible would follow the training so that a consistent manner of dealing with student mental health problems could be established. Another mentioned that it would be nice to see if other colleagues who hadn’t followed the training would approach colleagues who had and asked them for help in mental health-related issues, so that the knowledge and competencies would further spread within the school.

### Preliminary effectiveness

Table 3 shows the results on preliminary effectiveness, based on the pre-post comparison. Participants’ mental health literacy was significantly higher after having followed the intervention: Their self-rated awareness, knowledge, and comfort improved as well as their objectively measured knowledge and stigma. Almost all items were strongly significant (*p* < 0.001). However, the scores on the KAMHS domain of ‘self-stigma’ were not significantly higher after the intervention (*p* = 0.11). There was also no significant change in participants’ responses on the general items ‘Access for students to help resources at school needs to be better’ (*p* = 0.44) and ‘It is important that educational professionals have insights into the mental health problems that students can experience’ (*p* = 0.15). Finally, the participants indicated (on a scale from 0 to 4) that the training has adequately contributed to their increase of mental health literacy scores (Awareness: *M* = 2.46 (SD = 0.78) Knowledge (subjectively measured) *M =* 2.37 (SD = 0.77); Comfort *M* = 2.42 (SD = 0.83)).

**Table 3:** Comparison of mental health literacy scores of educational professionals before (pre) and after (post) intervention per subdomain (*N* = 29); scores ranging from 0 to 4 with higher scores indicating higher MHL

Item text	Pre M (SD)	Post M (SD)	t (df)	Cohen’s d	*p*
General items					
I am fully trained as a professional to deal with students who have a mental health problem or mental health disorder.^1^	0.99 (0.52)	1.65 (0.25)	−5.74 (28)*	−1.07	< 0.001
I am confident in my skills to identify mental health problems in students.	1.90 (0.98)	2.76 (0.87)	−4.38 (28)*	−0.81	< 0.001
Access for students to help resources at school needs to be better.	2.34 (1.01)	2.31 (1.00)	0.15 (28)	0.03	0.44
It is important that educational professionals have insights into the mental health problems that students can experience.	3.45 (0.51)	3.59 (0.57)	−1.07 (28)	−0.20	0.15
Awareness I am aware of…					
The range of mental health issues that children and youth experience during the school years.	1.35 (0.75)	2.62 (0.70)	−7.40 (25)*	−1.75	< 0.001
The risk factors and causes of student mental health issues.	1.38 (0.70)	2.62 (0.70)	−6.91 (25)*	−1.77	< 0.001
The types of treatments available to help students with mental health issues (e.g., counselling).	1.04 (0.82)	2.12 (0.86)	−5.20 (25)*	−1.29	< 0.001
The local community services for treating students with mental health issues (e.g., do you know who to call?).	1.31 (1.01)	2.50 (0.91)	−6.20 (25)*	−1.24	< 0.001
The steps necessary to access local community services for mental health issues.	1.91 (1.06)	2.27 (0.96)	−5.03 (25)*	−0.36	< 0.001
To what extent has the LEARN-NL training helped you increase your awareness of these issues?	-	2.46 (0.78)	-	-	-
Knowledge (subjectively measured) I am knowledgeable…					
About the signs and symptoms of student mental health issues.	1.23 (0.82)	2.77 (0.65)	−8.28 (25)*	−2.08	< 0.001
About appropriate actions to take to support student mental health at school	0.88 (0.77)	2.25 (0.83)	−6.65 (25)*	−1.71	< 0.001
About legislation related to mental health issues (confidentiality, consent to treatment, etc.).	0.85 (0.83)	1.96 (0.82)	−5.31 (25)*	−1.35	< 0.001
About school system services and resources for helping students with mental health issues.	1.00 (0.82)	2.27 (1.04)	−5.46 (25)*	−1.35	< 0.001
To what extent has the LEARN-NL training helped you increase your knowledge of these issues?	-	2.37 (0.77)	-	-	-
Comfort I feel comfortable…					
Talking with students about mental health.	1.96 (0.96)	2.81 (0.94)	−4.28 (25)*	−0.89	< 0.001
Talking with parents about their child’s mental health.	1.77 (0.91)	2.54 (0.86)	−4.32 (25)*	−0.87	< 0.001
Providing support to students with mental health issues.	1.77 (1.14)	2.58 (0.95)	−3.53 (25)*	−0.77	0.002
Accessing school and system services for students with mental health issues.	1.58 (1.07)	2.58 (0.99)	−4.37 (25)*	−0.97	< 0.001
To what extent has the LEARN-NL training helped you increase your comfort of these issues?	-	2.42 (0.83)	-	-	-
KAMHS subscales					
Knowledge about Mental Health subscale (objectively measured)	2.75 (0.29)	3.01 (0.40)	−4.17 (23)	−0.74	< 0.001
(Lack of) Self-Stigma subscale^1^	1.53 (0.16)	1.59 (0.15)	−1.23 (27)	0.39	0.11
(Lack of) Stigma subscale	2.87 (0.44)	3.08 (0.51)	−2.68 (27)*	0.44	0.006

* p-value = < 0.05; ^*1*^ *= data normalized through square root transformation*

## Discussion

In this study, we assessed the feasibility of a new mental health literacy intervention for Dutch educational professionals in terms of acceptability, implementation, and preliminary effectiveness. Our results showed that LEARN-NL is acceptable, can be implemented as planned, and has the potential to be effective for increasing educational professionals’ MHL.

The LEARN-NL intervention was perceived as acceptable by the educational professionals who completed the training, as it matches the needs of educational professionals and addresses topics and content that are essential to their professional tasks. Our findings show that educational professionals find LEARN-NL useful and relevant as it provides them with the necessary knowledge to develop further mental health skills and competencies to promote adolescents’ mental health and facilitate understanding. Other mental health literacy interventions for educators, such as the ‘Go-to’ Educator Training or a Haitian-based mental health educator training have also been rated as highly useful [37, 38]. There are currently no other evaluations available regarding the perceived usefulness of this specific intervention, indicating a need for further research in this area to validate our findings. The acceptability of LEARN-NL, as experienced by those who completed the training, highlights once more how important it is for educational professionals to be competent and knowledgeable on mental health topics in the school context, similar to findings by other studies [17, 39, 40]. This finding may be explained by the fact that the intervention was developed based on the previously identified need of educational professionals and adolescents of improved MHL of professionals [10] and was co-created with educational professionals to adapt the intervention to their specific needs [25]. This process may have led to a more acceptable intervention.

Our results show that the training contributes to improvements in knowledge of mental health, disorders, student stress management, and the capacities of participants to support adolescents. However, some learning outcomes of the training were less achieved, the intervention contributed to a lesser extent to educational professional personal stress management and insights into care processes. While the training’s impact on educators’ stress management may be deemed a secondary outcome, these findings imply room for improvement in these two facets. Future efforts may therefore want to improve the materials concerning the insights into care processes, by making the intricate details relating to youth mental health care (e.g., referral system, available mental health professionals) clearer to educational professionals.

With regard to the implementation of LEARN-NL, our findings show that the digital format of the training was most appreciated by the educational professionals. The preference for self-paced and location-independent training became evident, however time allocation was often challenging and there was a desire for more interaction. Previous research has identified comparable patterns in the professionalization methods of educational professionals, indicating that educational professionals in the 21 st century prefer digital or online professional development, where they have control over their learning in terms of time, place, or speed [41]. Combining digital learning methods with opportunities to interact and practice new skills with peers might be beneficial for MHL development among educational professionals [22, 42]. Future efforts may therefore invest in combining online education and in-person skills training for MHL interventions for educational professionals, by applying ‘blended learning’ methods [43].

Our results regarding preliminary effectiveness suggest that the LEARN-NL intervention can contribute to a better MHL of educational professionals in terms of improved mental health awareness, knowledge, and comfort, and reduced mental health stigma. These results are in line with the confirmed effectiveness of the original LEARN intervention that has shown equally improved mental health knowledge and attitudes of pre-service educators in large samples [20, 24]. While our results are largely promising regarding the improvement of MHL, we did not find an effect of the training on self-stigma. The reported evidence regarding the effects of MHL interventions on self-stigma is inconsistent, whereas evidence supporting their impact on public stigma is considerably more convincing [44]. Generally, decreasing self-stigma in adults can be a complex and challenging process as negative beliefs and attitudes regarding mental health and disorder have likely been internalized for several decades [45, 46]. Future research could explore which methods within MHL interventions are most effective in reducing self-stigma.

To summarize, our findings suggest that LEARN-NL could be a promising intervention to train educational professionals in promoting mental health at schools, identifying adolescents with mental health problems at an early stage, and supporting them by finding the right help sources at the right time, if needed. LEARN-NL has the potential to replicate the effectiveness of the original intervention in terms of enhancing knowledge, awareness, and comfort regarding addressing mental health (problems) of adolescents and reducing stigma. This should be evaluated in a larger-scale study.

### Strengths and limitations

A major strength of this study was its mixed-methods design which gave us important insights into the feasibility of the intervention from a quantitative and a qualitative perspective. These insights will stimulate the further refinement of the intervention.

However, some limitations ought to be addressed as well. This study did not include a control group with which we could compare the intervention effects. This limits the potential for inferences on effectiveness. Moreover, drop-out rates from the intervention were relatively high which have led to overestimation of the intervention effects and its feasibility. Participants who were able and willing to make time for the training, and who preferred self-paced (digital) learning, may have prioritized the training and thus were able to complete the training. Furthermore, only those who completed the intervention provided feedback on its acceptability and implementation of the training, potentially biasing the results. This self-selection effect suggests that the reported feasibility might not fully represent the broader population of educational professionals. Interviews with the non-completers could have provided valuable insights into the reasons for drop-out and thus acceptability and implementation. Additionally, the snowballing recruitment method may have introduced selection bias by over-representing more willing educational professionals. Future evaluations and implementation rounds of LEARN-NL should offer flexible scheduling options, use whole-school recruitment strategies, and provide ongoing support and incentives to improve completion rates. It is also recommended to actively investigate the reasons for drop-out and non-completion to better understand these issues and make more generalized conclusions about the intervention’s feasibility and effectiveness.

Finally, while our overall results are positive, they could partly be attributed to the so-called Hawthorne effect [47], i.e. an effect of the mere participation in this study. This is unlikely though, as objectively measured mental health knowledge did significantly increase following the intervention (p = < 0.001). This further substantiates the genuine impact of the LEARN-NL intervention.

### Implications

The findings of this study have several implications for practice. First, the results of this study can lead to improved implementation and uptake of LEARN-NL and therefore improved MHL of educational professionals. LEARN-NL may also be promising for enhancing MHL not only for active professionals, but also for those still in training. Implementing modules on mental health and MHL in teacher education programs may be an effective route of equipping future educational professionals with the necessary knowledge and skills in this area. Second, our results identified several factors that can aid the implementation of LEARN-NL and other intervention(s) in the school environment. A particular point of interest became visible in terms of digital learning methods for educational professionals which were well-received and perceived as useful to establish new theoretical knowledge. The findings of this study can aid future intervention developers and implementers in their plan of action when implementing and testing interventions with educational professionals and schools.

The implications for research are as follows: First, our findings indicate that LEARN-NL has the potential to improve MHL among Dutch educational professionals. Future research may investigate the long-term effectiveness of the intervention in a bigger sample and compared with a control group to further study the intervention’s robustness. Second, our findings may bring about cross-cultural research initiatives on the MHL of educational professionals. Our promising results may inspire other researchers to explore the MHL of educational professionals in other contexts and improve it with a version of LEARN adapted to their specific context.

## Conclusion

The LEARN-NL intervention appears to be feasible, in terms of acceptability, implementation, and preliminary effectiveness, to increase educational professionals’ mental health literacy in the Netherlands. This intervention has the potential to improve Dutch educational professionals’ mental health literacy, which might lead to more understanding and empathy for, and early identification of adolescents with mental health problems. Future studies may confirm these findings in a larger sample and provide evidence of effectiveness.

## Supplementary Information

Supplementary Material 1. Description LEARN-NL.

Supplementary Material 2. Qualitative Interview Protocol LEARN-NL.

Supplementary Material 3. Supplementary table 1. Main themes, subthemes and supporting quotes of participants regarding acceptability.

Supplementary Material 4. Supplementary table 2. Main themes, subthemes and supporting quotes of participants regarding implementation.

## Data Availability

The datasets used and/or analyzed during the current study are available from the corresponding author on reasonable request.

## Abbreviations

MHL: Mental health literacy
KAMHS: Knowledge and Attitudes to Mental Health Scales
MHLCSE: Mental Health Literacy and Capacity Survey for Educators
MOOC: Massive Open Online Course
MIDI: Measurement Instrument for Determinants of Innovations
EP: Educational Professionals
