# Supplementary material for: Improving mental health literacy of educational professionals: feasibility and preliminary effectiveness of an adapted intervention LEARN-NL
Source: BMC Public Health. 2025 Sep 30;25:3157. doi: 10.1186/s12889-025-23836-4 (PMC12487001; doi:10.1186/s12889-025-23836-4)
Supplement: Supplementary file 1 — Supplementary Material 1. Description LEARN-NL. [file 12889_2025_23836_MOESM1_ESM.docx]

**Qualitative Interview Protocol LEARN-NL**

**Respondent number:**

Date:

Start of participation in the study: <date>

1. **Acceptability**
2. What was it like to follow the training? Tell us about your experience.
3. What did you get out of the LEARN-NL training? (D7)
   1. How did you benefit from the training in terms of your general knowledge about mental health/identifying mental health problems/dealing with students with mental health problems? (D7)
   2. Which specific learning formats of the training did you like the most/least?
      1. *Activities, theory, reflection?*
   3. What knowledge and/or skills would you have liked to learn?
      1. *Signaling? Dealing with students with mental health problems? General knowledge about mental health problems?*
4. Would you recommend the training to your colleagues or those from other schools? Why?

*(Suppose the Ministry of Education thinks that LEARN-NL should be followed by every education professional...)*

- 1. *Why or why not?*
  2. What can the training do for teachers in terms of mental health literacy?

1. What does the LEARN-NL training bring to your students? How do students benefit from your participation in this training? What does the training mean for your students? (D7)
   - 1. *What specific parts make it valuable/less valuable?*
   1. Do the skills learned in LEARN-NL match the needs of secondary school students?

*Age/adolescent brain/different school levels*

1. **Implementation**
2. What did you think of the form of the training (individual, digital)?
   1. What are the benefits?
   2. What are negatives?
   3. What did you think of the goals and the instructions to be able to follow the training? (D1)
      1. Broad? Clear?
   4. Did you follow all the modules of the training? Which ones do, which ones don't?
      1. *Have you completed all the activities LEARN-NL? Which ones do, which ones don't?*
      2. *If some don't, why not*
   5. To what extent were you able to combine the training with your other activities/teaching activities?
      1. What role did your school's management play in being able to follow the training?
   6. To what extent can the training be combined with the usual tasks of a teacher?
      1. *E.g. digital version versus physical course*
3. What new insights/knowledge and skills did you gain in the training?
   1. How would you like to apply your new insights/knowledge + skills in the future?
      1. Why yes, why not?
   2. How do you think you can apply your new insights/knowledge + skills in practice? (D16)
      1. If not, why not?
   3. Are there any other changes within the school that you've had to deal with that affected the use of the skills from LEARN-NL? (D26)

*For example: reorganization, budget cuts, staff turnover, absenteeism, etc.*

1. Other
   1. Are there any other comments you would like to make about the training?
