# Supplementary material for: Improving mental health literacy of educational professionals: feasibility and preliminary effectiveness of an adapted intervention LEARN-NL
Source: BMC Public Health. 2025 Sep 30;25:3157. doi: 10.1186/s12889-025-23836-4 (PMC12487001; doi:10.1186/s12889-025-23836-4)
Supplement: Supplementary file 2 — Supplementary Material 2. Qualitative Interview Protocol LEARN-NL. [file 12889_2025_23836_MOESM2_ESM.docx]

| **Supplementary table 2.** Main themes, subthemes and supporting quotes of participants regarding implementation. | |
| --- | --- |
| **Main themes and subthemes** | **Supporting quotes** |
| ***Format*** | |
| Well-received digital format | *“Doing it online was only positives for me, at home I’m by myself, I have a double screen. I wouldn't be able to focus if I had a lot of people around me, then I really have to put on headphones, so I really liked it and I could do it in my own time.”* (EP5)  *“It does require a certain discipline because you must organize it yourself. But of course, it also has a lot of added value that you can plan it yourself, I do think that more people are able to participate.”* (EP9) |
| Combination with daily activities | *"Otherwise I wouldn't have been able to do this course like this now, because I have to do it in my own time. I have to be able to schedule it when I can and that's the disadvantage of physical meetings, everyone has to be physically together at a specific time."* (EP3).  *“I did have room to do [the training] in between, but if you have a busy class schedule, yes, then you either have to do it at the end of the day or on your day off and then it becomes a lot more difficult.”* (EP6). |
| Missing peer interaction | *"The downside was that I was only able to have the conversation about this at the coffee machine and if you had done this at school in a classroom and then I think you would have sparked the conversation more. Now it has to be done from your own intrinsic motivation."* (EP4)  *“You [can never compare], or that you can talk about with someone else. How did you do that? In a physical training you also have your colleagues who are at the same point with you, so they run into the same things, have the same questions, but from whom you can also learn something, I missed that now because it was all online.”* (EP6) |
| ***Conditions for broad implementation and impact*** | |
| Applicable to different target groups | *"Of course, the best thing would be if you also made these things known at a teacher training program, that you offer this as a kind of minor, for example."* (EP4)  *“Often a school management is a group of people who remotely manage a school and […] if they are not informed and if they do not know what is going on in the school, if they don’t even have a look at such a training, then I think you may miss the necessary input as a school board.”* (EP4).  *“I think it is particularly valuable for teachers who have little knowledge of this. [...] And with the introduction of the Inclusive Education Act. But what other training is there for it?”* (EP8) |
| School-wide implementation | *"I hope that the colleagues who have not followed the training will come and ask questions to us who have followed the training, so that it will spread like a fire through the school."* (EP4)  *"I hope that we will continue to talk about this with each other, that we discuss with this knowledge, that we will now also look that way at students who are dealing with it [mental health problems], so that we can apply things in the interest of the students. It should not be the case that it becomes diluted."* (EP4)  *"I hope there will be more of a consistent way in how we are going to take care of and support students."* (EP8). |
| ***EP = Educational professional*** | |
