# Supplementary material for: Improving mental health literacy of educational professionals: feasibility and preliminary effectiveness of an adapted intervention LEARN-NL
Source: BMC Public Health. 2025 Sep 30;25:3157. doi: 10.1186/s12889-025-23836-4 (PMC12487001; doi:10.1186/s12889-025-23836-4)
Supplement: Supplementary file 3 — Supplementary Material 3. Supplementary table 1. Main themes, subthemes and supporting quotes of participants regarding acceptability. [file 12889_2025_23836_MOESM3_ESM.docx]

| **Supplementary table 1.** Main themes, subthemes and supporting quotes of participants regarding acceptability. | |
| --- | --- |
| **Main themes and subthemes** | **Supporting quotes** |
| ***Participant satisfaction*** | |
| New useful information | *"I really have more tools now to hopefully know how to approach the students. [...] So my equipment has been refreshed and replenished."* (EP5)  *"I thought it was very interesting to do because what you read is very much in line with what you see in the classroom. But what you also see among colleagues, in your own environment, friends, acquaintances, relatives.”* (EP6) |
| Practical tips and tools | *“In education, you come across a lot of students who you feel like aren’t doing well. But to warn parents or discuss them with students, how do you do that? How do you approach that?”* (EP6)  *"How do you respond if a student comes to you like, "I can't handle it." That you know how to put that in perspective. That ability to put things into perspective, and that course has contributed to that."* (EP3) |
| Not learned previously | *"[This] didn't come up at all [in my education]. I was trained to become a physical education teacher and then you learn a lot about social emotional growth, but not about mental health at all."* (EP6)  *“When I saw [the training], I thought, yes, this is something that I always feel like, hey, I'm not very good at this, so everything I can learn from this is a bonus.”* (EP7) |
| Less useful information | *"Most of the kids I deal with have already been diagnosed with ADHD or ADD or things like that. That's already in the files for us.”* (EP2).  *"[The less interesting parts were] perhaps also a repetition of what you already knew. [..] For example, about support because I also do quite a bit with support here at school and I have been a mentor for quite a few years."* (EP6) |
| Recommendations about the content | *"Information on behavioral problems, especially aggression from students and abusive language, and how to deal with that, would have been valuable. [..] I often think yes, I've solved it, but actually, I don't feel good about it myself."* (EP8) |
| ***Rich diversity of topics covered*** | |
| Stress response and management | *"That was very useful for me at school as a mentor because all children at our school are experiencing a lot of stress right now [...]. And that you can also turn stress into the positive, I personally liked that the most."* (EP1)  *“Because I'm now more alert to the fact that I'm going to say "It's not a big deal, stress isn't a big deal" during tests. Whereas in the past, we were more likely to be involved when students experience stress when preparing for the test, for example, we do test anxiety reduction training. Well, I think differently about that now."* (EP3) |
| Specific mental disorders | *“In the module, all disorders were also described, [...] I found that very interesting, because then you see hey what is it, what happened to it and how can you deal with it? What did help? That actually gives a lot of information.”* (EP6)  *“One of the most important things that has stayed with me is the difference between just not feeling good mentally and mental disorders.”* (EP7) |
| Stigma reduction | *“Then you come back to that part of the stigma that you change from ‘It's crazy, it's weird, you're no good’ to ‘everyone can have something, and one suffers from this, the other suffers from that. That's not weird at all’."* (EP1)  *"The stigma part, you honestly don't think about it. That was a real eye-opener again.”* (EP4) |
| ***Better mental health literacy of educational professionals aiding adolescents*** | |
| More understanding | “*I hope that there will be more understanding for the young people and that we support the young people in their capacities, their opportunities, tailored to their needs, in thinking along, so being empathetic towards students without stigmatizing, and just having an open and honest conversation and staying connected with the young people.”* (EP4).  *"It means for the students that they feel safer and more understood and that it is easier to get the right help. [..] It's nice for them that the number of teachers [that can support them] is getting bigger and that there is a listening ear."* (EP6) |
| Adolescent empowerment | *“I want to teach them how to deal with stress and so I think we can play a very important role in that as teachers.”* (EP1)  *“For example, we have a test anxiety reduction training. And what you're going to do after that is actually trying to reduce that fear. But you can also talk someone through that fear, just explain what it's for and why your body reacts the way it does. Well, that was an eye-opener for me, I thought that was a very nice one.”* (EP9) |
| Problem identification and referral to help services | *"Well, I think you're more alert to notice when a student starts to change."* (EP5)  *"I think through early detection, there may be less of a problem, or that it is less likely to scale up to external help, but that mentors themselves is better equipped [through this training] to offer low-threshold support."* (EP9) |
| ***Note: EP = Educational professional*** | |
