# Supplementary material for: Improving mental health literacy of educational professionals: feasibility and preliminary effectiveness of an adapted intervention LEARN-NL
Source: BMC Public Health. 2025 Sep 30;25:3157. doi: 10.1186/s12889-025-23836-4 (PMC12487001; doi:10.1186/s12889-025-23836-4)

**Description LEARN-NL**

The training is designed as a comprehensive digital self-study course that offers participants an opportunity for reflection and engagement through interactive activities, videos, and practical tools and tips. It is structured into six modules, each addressing crucial aspects of mental health literacy and mental health support for adolescents in secondary education:

Module 1: Basic Concepts on Mental Health Literacy and Mental Health

This module lays the foundation by introducing key concepts related to mental health literacy and understanding the importance of mental health awareness in an educational setting.

Module 2: Understanding Stress and the Stress Response

Participants learn about stress and its impact on adolescents, equipping them with knowledge to recognize signs of stress and its potential effects on mental well-being.

Module 3: Stigma of Mental Health Disorders

This module addresses the issue of stigma surrounding mental health disorders and empowers educators to create a more inclusive and supportive environment for students experiencing mental health challenges.

Module 4: The Adolescent Brain

Understanding the unique developmental characteristics of the adolescent brain is crucial for educational professionals to effectively support and communicate with their students.

Module 5: Most Common Mental Health Disorders of Adolescents

This module provides insights into the most prevalent mental health disorders among adolescents, enabling participants to identify potential signs and symptoms in their students.

Module 6: Seeking Help and Providing Support

The final module equips participants with strategies for effectively communicating with adolescents and caregivers about mental health concerns. It also guides them in offering early-on mental health support and making appropriate referrals to available resources.

Overall, the training's primary objective is to equip educational professionals with the necessary competencies to recognize and address mental health problems in adolescents.


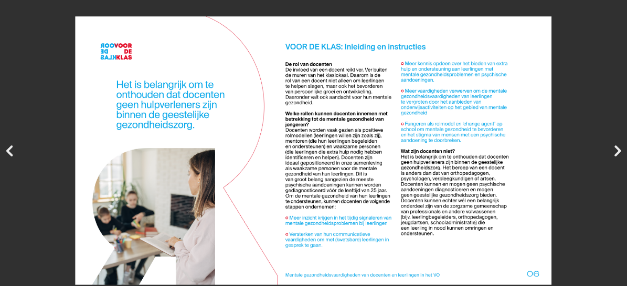

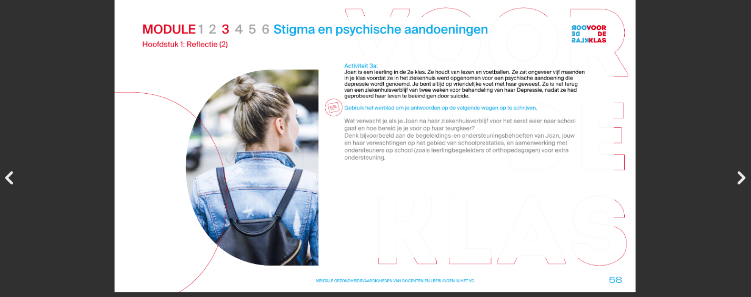


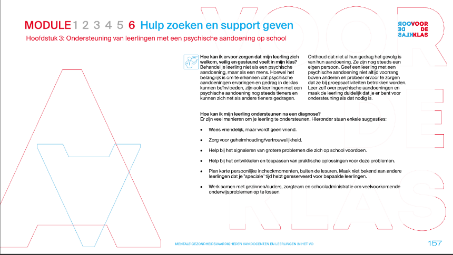

Supplement: Supplementary file 4 — Supplementary Material 4. Supplementary table 2. Main themes, subthemes and supporting quotes of participants regarding implementation. [file 12889_2025_23836_MOESM4_ESM.docx]
